# HPLC‐DAD‐ESI‐MS/MS Analysis and Evaluation of Antioxidant and Antidiabetic Activities of *Vachellia tortilis* subsp. *raddiana* (Savi) Kyal. & Boatwr. Extracts

**DOI:** 10.1002/fsn3.72360

**Published:** 2026-11-05

**Authors:** Douae Taha, Souad El Hajjaji, Yassine Mourabit, Waleed Al Abdulmonem, Mohammed Alorini, Abdelhakim Bouyahya, Naima Iba, Ilhame Bourais

**Affiliations:** ^1^ Laboratory of Spectroscopy, Molecular Modeling Materials, Nanomaterials Water and Environment – CERNE2D, Faculty of Sciences Mohammed V University in Rabat Morocco; ^2^ Department of Pathology College of Medicine, Qassim University Buraydah Saudi Arabia; ^3^ Laboratory of Human Pathologies Biology, Faculty of Sciences Mohammed V University in Rabat Morocco; ^4^ Department of Pharmacy Practice, Faculty of Pharmacy Universitas Airlangga Surabaya Indonesia

**Keywords:** antihyperglycemic potential, antioxidant, HPLC‐DAD‐ESI‐MS/MS, phenolic compound, *Vachellia tortilis* subsp. *raddian*

## Abstract

The current study examined the phytochemical composition and biological activities of *Vachellia tortilis* subsp. *raddiana*, a medicinal plant that remains understudied in Morocco. The antioxidant and antihyperglycemic activities of hydroethanolic leaf extract and aqueous gum extract were assessed; the two extracts markedly differed in their polyphenolic compound, with the leaf extract showing higher levels of total phenols (40.381 ± 0.124 mg GAE/g DW), flavonoids (71.351 ± 0.233 mg RE/g DW), and tannins (3.211 ± 0.021 mg CE/g DW). In the DPPH, ABTS, and FRAP assays, this higher phenolic content was associated with stronger antioxidant activity (IC_50_ = 0.032 ± 0.002 mg/mL; IC_50_ = 0.177 ± 0.002 mg/mL; and 42.783 ± 0.301 mg TE/g DW, respectively), whereas the gum extract demonstrated lower antioxidant capacity. Additionally, a stronger antihyperglycemic potential was suggested by the higher α‐amylase inhibitory activity of the hydroethanolic leaf extract (IC_50_ = 0.379 ± 0.002 mg/mL) compared with the aqueous gum extract (IC_50_ = 0.461 ± 0.001 mg/mL). Several bioactive chemicals were found in both extracts by phytochemical analysis using HPLC‐DAD‐ESI‐MS/MS, which could be responsible for the biological activities that were observed. Overall, these findings highlight the potential of *V. tortilis* as a source of natural compounds with antioxidant and antidiabetic properties. Further investigations are required to better understand the mechanisms involved and to support its possible therapeutic applications in the management of hyperglycemia.

## Graphical abstract

The study investigated the phytochemical composition of *Vachellia tortilis* subsp. *raddiana* leaf and gum extracts using HPLC‐DAD‐ESI‐MS/MS and evaluated their antioxidant and antidiabetic activities. The leaf extract showed a higher content of phenolic and flavonoid compounds and exhibited promising antioxidant and α‐amylase inhibitory activities.

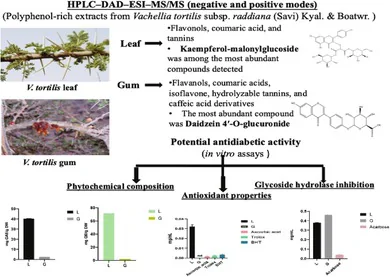

## 1 Introduction

For centuries, plants have been essential in the treatment of several illnesses (El‐Hilaly et al. 2003). In recent times, medicinal plants have experienced increased interest as valuable sources of novel medications due to their wide variety of bioactive chemicals, especially secondary metabolites including terpenoids, tannins, alkaloids, and flavonoids (Najmi et al. 2022; Huang and Zhang 2022). These bioactives have demonstrated many pharmacological properties, making them essential in the development of new therapeutic agents (El‐Hilaly et al. 2003).

β‐cells dysfunction, a hallmark of diabetes, resulted from the decline of antioxidant stress, inflammation, and impaired β‐cell neogenesis. Oxidative stress led to the disruption of cellular homeostasis, leading to the activation of several signaling pathways, including p38 MAPK, JNK/SAPK, NF‐kB, and hexosamine. These pathways collectively contribute to β‐cell dysfunction and apoptosis, highlighting the need to target them for effective diabetes prevention and management (Ou et al. 2025).

Multiple studies have revealed the anti‐diabetic effects from natural remedies including amylase enzyme inhibitors, which may reduce insulin levels by slowing the absorption rate of carbohydrates (Asanga et al. 2025; Bourais et al. 2023). In Addition, several studies that the plant extracts, due to their high antioxidant content, can act as effective amylase inhibitors (Bouyahya et al. 2017; Khadayat et al. 2020; Sharma et al. 2022; Bourais et al. 2022).

*Vachellia tortilis* subsp. *raddiana* (Savi) Kyal. & Boatwr., locally known as “Talh”, is a prominent species in Moroccan traditional medicine. It is synonymous with *Acacia tortilis* subsp. *raddiana* (Savi) Brenan and has been traditionally used to treat a number of disorders, most notably stomach aches and diabetes (Benkhnigue et al. 2014; Ghourri et al. 2013; Taha, Bourais, et al. 2022; Taha, El Hajjaji, et al. 2022). In Moroccan ethnopharmacology, *Vachellia* is recognized as one of the most frequently used plants in traditional applications (Hammiche and Maiza 2006; Al‐Fatimi et al. 2007; Taha, El Hajjaji, et al. 2022). Pharmacological studies have documented its diverse properties, notably cytotoxic activity (Alajmi et al. 2017; Koch et al. 2005), antileishmanial activity (Kigondu et al. 2009), anticonvulsant effects (Alharbi et al. 2011), antimalarial activity (Koch et al. 2005), antimicrobial activity (Ali and El Said 2020; Abdllha et al. 2016; Alajmi et al. 2017; Al‐Fatimi et al. 2007; Sulayli et al. 2019), and antifungal activity (Abdllha et al. 2016; Alajmi et al. 2017; Al‐Fatimi et al. 2007; Sulayli et al. 2019).

The curious therapeutic potential of *V. tortilis* in the treatment of both infectious and chronic disorders is highlighted by its high amount of bioactive chemicals that demonstrate notable antibacterial, antioxidant, and anti‐inflammatory effects. Additionally, the variety of its phytochemical components is an important resource for pharmaceutical research, especially when it relates to antibacterial and anticancer treatments (Al‐Rajhi et al. 2026; Herchi et al. 2025; Mokhtar et al. 2025).

Thus, the current work focuses on organic leaf extracts and aqueous extracts of lyophilized gum in order to examine the phytochemical profile of *V. tortilis* using HPLC‐DAD‐ESI‐MS/MS analysis. Additionally, utilizing DPPH, ABTS, and FRAP assays to measure total phenolic, flavonoid, and condensed tannin concentrations as well as antioxidant activities, the study assesses its biological potential. Using α‐amylase inhibitory activity, the antidiabetic potential was evaluated. The aim of this work is to present a thorough understanding of the connection between bioactivity and chemical properties. To the best of our knowledge, this work supports this subspecies' prospective uses in the pharmaceutical and nutraceutical industries by providing new insights into its biological traits and chemical characterization, especially in the Moroccan environment.

## 2 Material and Methods

### 2.1 Plant Material

During the dry season (July 2020), *V. tortilis* leaves and gum were harvested from laayoune, and oued ighzi located in the Sahara region along the atlantic coast of morocco (latitude: 66 m). The plant material was taxonomically identified, and its identity was confirmed with a voucher specimen (RAB 111532) by Dr. Hamid El Khamar of the Botany Department, University of Mohammed V, Rabat, Morocco.

### 2.2 Preparation of Extracts

#### 2.2.1 Preparation of the Aqueous Extract

Briefly, 30 g of gum powder was macerated in 300 mL of distilled water for 24 h. After filtering the combination, the residue was purified at 80°C using rotary evaporators. The concentrate was subsequently lyophilized into a fine powder to remove residual water. The final product was stored at −20°C until extra analysis.

#### 2.2.2 Preparation of the Hydroethanolic Extract

Thirty grams of leaf powder was macerated in 300 mL of 80% ethanol at room temperature with continuous stirring for 24 h. The mixture was filtered, and the filtrate was concentrated using a rotary evaporator to remove the solvent. The resulting extract was stored at 4°C until usage.

### 2.3 Determination of Phenolic Contents

#### 2.3.1 Total Phenolic Content

Folin Ciocalteu reagent method was used to determine the Total Phenolic Content (TPC), as described by Tian et al. (2019) with minor adjustments. Gallic acid solutions in concentrations ranging between 10 and 300 μg/mL were used to prepare a standard. Initially, 2.5 mL of the sample solution was mixed with 4 mL of 7.5% (w/v) Na_2_CO_3_. At 45°C, the reaction was permitted to continue for a period of 15 min. Milligrams of gallic acid equivalents (GAE) per gram of dry weight of extract (mg GAE/g DW) were used to calculate TPCs. A blank was used as a reference when measuring the absorbance at 765 nm.

#### 2.3.2 Total Flavonoid Content

Total Flavonoid Content (TFC) was assessed by Brighente et al. (2007) method, a colorimetric technique. Briefly, an aliquot of the extract or standard at various concentrations was added to a test tube. Then, 0.2 mL of 10% AlCl_3_ solution and 0.02 mL of 1 M CH_3_CO_2_K were added. The reaction mixture was then diluted with 5.6 mL of distilled water. The mixture was then incubated for 30 min at 25°C. At 510 nm, the absorbance was measured.

#### 2.3.3 Total Tannin Content

Total Tannin Content (TTC) was assessed using Prayogo's technique (Prayogo et al. 2021), which is used to calculate the amount of condensed tannins. Briefly, 0.75 mL of HCl (12 M) was added to the sample, followed by the addition of 50 μL of the sample solution to 1.5 mL of vanillin dissolved in methanol. Catechin concentrations ranging from 20 to 1000 μg/mL were used to create a standard curve.

### 2.4 HPLC‐DAD‐ESI‐MS/MS Analysis

#### 2.4.1 HPLC‐DAD‐ESI‐MS/MS Analysis of Aqueous Extract

The High‐Performance Liquid Chromatography (HPLC) system used was coupled with Diode‐Array Detection (DAD) and ElectroSpray Ionization‐tandem Mass Spectrometry (ESI‐MS/MS) for the analysis of aqueous extract. The UHPLC system was equipped with a photodiodes array and a mass system with a waters acquity UPLC PDA detector, at a fixed wavelength of 500 nm. The analysis was controlled by masslynx software (v4.1). The extract was dissolved in methanol and separated on a 150 × 1 mm, 1.7 μm, UPLC C18 column, maintained at 35°C. The mobile phase consisted of two solvents: A (H_2_O + 10% CH_3_CN + 1% CH_3_COOH) and B (H_2_O + 80% CH_3_CN + 1% CH_3_COOH). For peak characterization, UV spectra were recorded from 190 to 500 nm.

The high‐resolution LC‐QTOF‐ESI‐HRMS analysis is carried out using an agilent 6530 mass spectrometer. Mass spectra were acquired in both positive and negative ion modes over a mass‐to‐charge ratio (*m/z*) range of 100–3000, using electrospray ionization coupled with a time‐of‐flight (TOF) mass analyzer. The flow rates for the cone gas and the desolvation gas were 11 and 8 mL/min, respectively. The source temperature was maintained at 300°C, while 375°C was selected as the desolvation temperature.

#### 2.4.2 HPLC‐DAD‐ESI‐MS/MS Analysis of Organic Extract

For the analysis of the organic extract, two mobile phases (A and B) were used in gradient mode. Phase A consisted of water and acetic acid (999:1 v/v) while phase B was acetonitrile. The gradient program was as follows: Phase B started at 5% for the first five min, followed by a linear increase to 100% at 65 min. Chromatographic peaks were processed using MassLynx software. Compound identification was based on retention times and mass spectral data (*m/z*), which were compared with literature data for tentative identification.

The MS analysis was performed under the same conditions described for the aqueous extract.

### 2.5 Evaluation of the Antioxidant Activity

#### 2.5.1 DPPH Radical Scavenging Assay

The extracts' potential to scavenge free radicals was assessed using the stable radical DPPH, as determined by Katanić Stanković et al. (2020), A variety of the extracts were mixed with a freshly prepared DPPH solution (0.5 mL of DPPH dissolved in methanol). The mixture was left to stand at room temperature for 30 min. Ascorbic acid, Trolox, and BHT were used as reference compounds. The antioxidant activity of our extracts is expressed as the percentage of DPPH radical inhibition, and the IC50 was calculated for comparing the obtained results. The percentage of scavenging activity was expressed as:

(1) $$\text{DPPH scavenging effect}\;\left(\%\right)=\frac{A_{0}-A_{1}}{A_{0}}\times 100$$

where 𝐴_0_: absorbance of the control and 𝐴_1_: Absorbance of the sample solution or standard.

#### 2.5.2 ABTS Radical Scavenging Assay

The blue green ABTS was generated by combining 2 mM ABTS with 70 mM K_2_S_2_O_8_ in water. Trolox was used as a reference compound. Calculating the proportion of scavenging activity was done using formula (1) (Wu et al. 2020). The absorbance was measured at 734 nm after combining 2 mL of diluted ABTS solution with 100 μL of plant for 1 min at room temperature.

#### 2.5.3 Ferric Reducing Antioxidant Power Radical Scavenging Activity

The ferric ions (Fe^3+^) reducing capacity was measured in accordance with (Tundis et al. 2013), with a few changes. In brief, 1 mL of extract was combined with 1.25 mL (1%) of potassium ferricyanide (C_6_FeK_3_N_6_) and sodium phosphate buffer (pH 6.6). For 20 min, the mixture was heated in a water bath at 50°C for 20 min. After cooling, 10% trichloroacetic acid (C_2_HCl_3_O_2_) was added, and the mixture was then centrifuged at  2000 g for 10 min. Lastly, 1.25 mL of the supernatant was combined with 1.25 mL of distilled water and FeCl_3_ solution. At 700 nm, the absorbance was obtained.

### 2.6 In Vitro Antidiabetic Activity of α‐Amylase Inhibition Assay

To prepare the substrate, 200 mg of starch was dissolved in 25 mL of 0.4 M NaOH and heated to 100°C for 5 min. Following cooling, a 20 mM phosphate buffer (pH 7.0) containing 6.7 mM NaCl was used to dilute the solution to a final volume of 100 mL. After that, the pH was elevated to 7.0.

Acarbose was utilized as a positive control. For the assay, 100 μL of acarbose or the test samples at various concentrations were pre‐incubated at 37°C for 15 min. Then, 200 μL of starch solution was added, and the mixture was incubated at 37°C for another 15 min. After adding 400 μL of HCl (0.1 M) and 500 L of iodinated reagent (2.5 mM), the reaction was stopped. The absorbance was measured at 630 nm. The percentage of inhibition was determined using formula (2) (Kusano et al. 2011).

(2) $$\begin{array}{c} \%\text{of inhibition}=\\ \frac{\left(\mathrm{Abs}\;\mathrm{enz}+\mathrm{sub}-\mathrm{Abs}\;\mathrm{sub}\right)-\left(\mathrm{Abs}\;\text{sample}-\mathrm{Abs}\;\text{control}\;\right)\;}{\left(\mathrm{Abs}\;\mathrm{enz}+\mathrm{sub}-\mathrm{Abs}\;\mathrm{sub}\right)}\times 100 \end{array}$$

where “Abs enz + sub” and “Abs sub” represent the positive control (substrate with enzyme), “Abs sub” and “Abs sample” indicate the negative controls' absorbance (substrate without enzyme), and “Abs control” refers to the substrate and sample without enzyme.

### 2.7 Statistical Analysis

All extracts were prepared once, and all analytical measurements were performed as technical replicates. Data are presented as mean ± standard deviation (SD) (*n* = 3 for phytochemical and antioxidant assays and *n* = 6 for the α‐amylase inhibition assay). Comparisons between the leaf and gum extracts were interpreted descriptively. Where appropriate, statistical comparisons among the reference standards were performed using one‐way ANOVA followed by Tukey's multiple comparisons test in GraphPad Prism 10. IC_50_ values were calculated by plotting the percentage inhibition against extract concentration and fitting the data using nonlinear regression (log(inhibitor) vs. normalized response − variable slope) in GraphPad Prism 10.

## 3 Results and Discussion

According to Table 1 and Figure 1, the phytochemical analysis revealed differences in the phenolic composition between the two extracts in terms of Total Phenolic Content (TPC), Total Flavonoid Content (TFC), and Total Tannin Content (TTC). For clarity, OLE (leaf extract) and AEGL (gum extract) are represented by L and G, respectively. Extract L exhibited markedly higher concentrations of phenolic compounds (40.381 ± 0.124 mg GAE/g DW) compared with extract G (2.396 ± 0.035 mg GAE/g DW) (Table 1). Similarly, the flavonoid content was considerably higher in extract L (71.351 ± 0.233 mg RE/g DW) than in extract G (1.916 ± 0.015 mg RE/g DW) (Table 1).

**TABLE 1:** Total phenolic, flavonoid, and tannin contents of *Vachellia tortilis* extracts.

Extracts	Yield of extraction %	TPC (mg GAE/g DW)	TFC (mg RE/g DW)	TTC (mg CE/g DW)
OLE (L)	23	40.381 ± 0.124	71.351 ± 0.233	3.211 ± 0.021
AEGL (G)	5.2	2.396 ± 0.035	1.916 ± 0.015	nd

*Note:* Data are expressed as mean ± SD (*n* = 3).

Abbreviations: AEGL, aqueous extract of lyophilized gum; CE, catechin equivalent; (G), gum; GAE, gallic acid equivalent; (L), leaves; nd, not detected; OLE, organic leaves extract; RE, rutin equivalent; TFC, total flavonoid content; TPC, total phenolic content; TTC, total condensed tannins.

**FIGURE 1:**
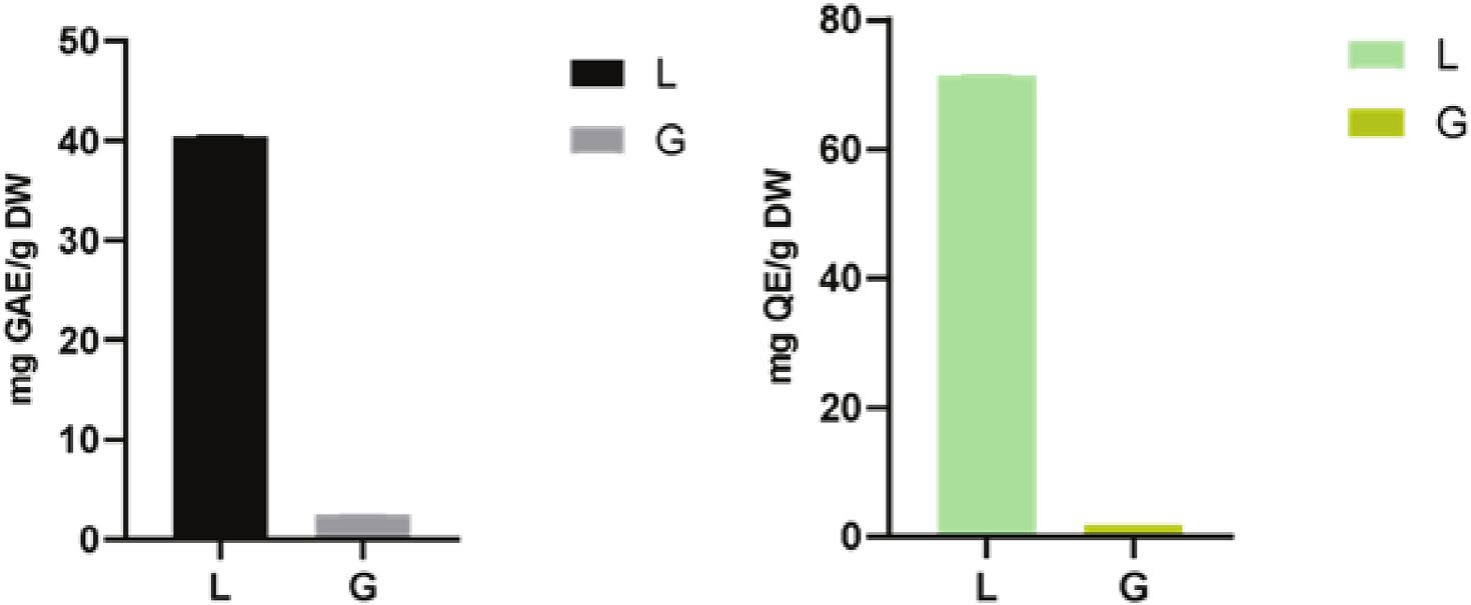
Comparison of total polyphenol and total flavonoid contents between the two extracts. OLE (leaf extract) and AEGL (gum extract) are represented by L and G, respectively.

The hydroethanolic leaf extract exhibited higher TPC, TFC, and antioxidant activity than the aqueous gum extract. However, these observations should be interpreted descriptively because the extracts differ in both plant organ and extraction solvent. Since the extracts originated from different plant organs and were prepared using different extraction solvents, the observed differences are presented descriptively rather than as direct statistical comparisons.

In addition, tannins were detected only in extract L (3.211 ± 0.021 mg CE/g DW), whereas they were not detected in extract G. The observed variation may also be related to the natural biochemical composition of the plant organs; leaves are known to be metabolically active tissues rich in secondary metabolites, whereas gum exudates comprise mainly of polysaccharides. These differences may be primarily explained by the polarity of the extraction solvent: hydroethanolic preparations generally improve the extraction of a larger variety of phenolic compounds, especially moderately polar flavonoids and tannins. While gum exudates are primarily made up of polysaccharides, leaves have been shown to be metabolically active tissues rich in secondary metabolites.

The hydroethanolic leaf extract of *V. tortilis* revealed higher phenolic and flavonoid levels than those reported for species including *Acacia saligna* , *Acacia cyclops* , and *Acacia mearnsii* when compared to earlier research on related species. These species had TFC values of 0.85 and 0.5 mg RE/g DW, respectively, and TPC values at 0.9 mg GAE/g DW (Buttner et al. 2021). Similarly, the TFC value of *Acacia nilotica* hydroethanolic extract was 37.531 ± 0.821 mg RE/g DW (Bilal Sadiq et al. 2015). A TFC value of 43.521 ± 0.141 mg RE/g DW was observed in another investigation on *V. tortilis* (Alphonsine et al. 2019).

Secondary metabolite accumulation has been demonstrated as impacted by several variables. Therefore, the observed variations should be interpreted as an indicator of the heterogeneity of phenolic biosynthesis under various ecological and methodological contexts rather than as absolute variations (Biswas et al. 2026; Cariño‐Cervantes et al. 2026; Stolecka and Gruszecka‐Kosowska 2026).

In experiments, a number of distinct phenolic compounds found in *V. tortilis* preparations have been directly demonstrated to have antidiabetic benefits. As reported in Figure 2 and Table 2, the HPLC‐DAD‐ESI‐MS/MS analysis of the hydroethanolic leaf extract identified several phenolic constituents, including quercetin 3‐O‐rutinoside (Rt = 17.26 min; *m/z* 609) and kaempferol‐malonylglucoside (Rt = 37.18 min; *m/z* 533). Two flavonols included in the extracts, quercetin and kaempferol (Figure 2), have shown remarkable antidiabetic properties in animal models by improving blood glucose levels and enhancing indicators of diabetic pathology. In streptozotocin‐nicotinamide‐induced diabetic mice, quercetin and kaempferol treatment substantially decreased hyperglycemia, improved lipid profiles, and decreased α‐amylase functions in vitro (Ali et al. 2024).

**FIGURE 2:**
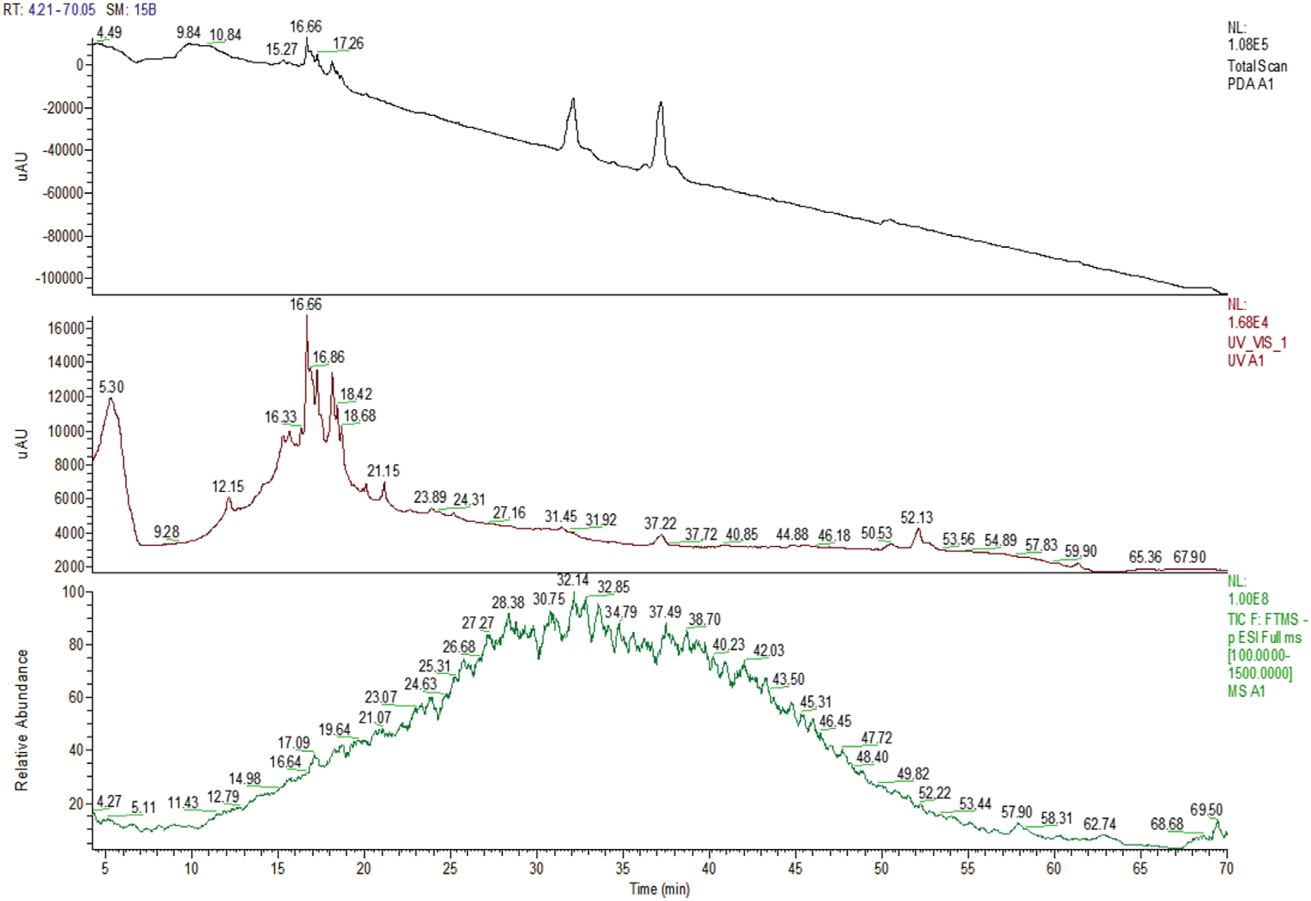
OLE sample showing the LC‐DAD total chromatogram recorded between 190 and 400 nm, the LC‐DAD chromatogram recorded at 280 nm, and the total ion chromatogram (TIC) acquired in negative ion mode.

**TABLE 2:** Identification of phenolic compounds of OLE sample by HPLC‐DAD‐ESI‐MS/MS.

No	Rt (min)	[M−H]^−^	MS/MS fragments (*m/z*)	Compounds	Chemical class
1	9.84	549	113/179/329/445/549	p‐Coumaroyl derivative	Coumaric acid
2	15.64	989	331/463/479/609/625/869/989	Quercetinrhamnosyl‐diglucoside	Flavonols
3	16.66	625	173/377/479/625	Quercetindiglycoside	Flavonols
4	16.87	577	233/377/447/479/577	Procyanidin B3	Anthocyanidines
5	17.26	609	193/479/609	Quercetin 3‐O‐rutinoside	Flavonols
6	18.13	639	121/255/281/303/595/639	Quercetin glucosyl‐glucuronide	Flavonols
7	18.42	591	463/493/561/591	Acacetina‐7‐O‐rutinoside (linarin)	Flavones
8	18.67	463	187/463	Ellagic‐glucoside	Tannins
9	25.37	385	145/187/195/233/385	Elenolic acid	Carboxylic acids
10	32.12	533	113/145/191/435/489/533	Dicaffeoylhexaric acid	Caffeic acid derivatives
11	37.18	435	293/316/343/385/435/533	Kaempferol‐malonylglucoside	Flavonols

In streptozotocin‐induced diabetic mice, kaempferol alone has been demonstrated to enhance glycemic control, certainly through improving peripheral tissue glucose use and regulating hepatic glucose synthesis (Alkhalidy et al. 2018). Additional evidence of quercetin's antidiabetic properties was provided by its treatment of STZ‐induced diabetic rats, which improved fasting blood glucose levels, restored insulin production, and decreased oxidative stress and inflammation indicators (Rahmani et al. 2023). When considered collectively, these findings imply that substances found in *V. tortilis* extracts, including quercetin and kaempferol, may alter important pathways related to glucose metabolism. The extracts' suspected antidiabetic qualities are supported by their effects, which may include enhanced glucose regulation in diabetic animals and inhibition of digestive enzymes.

Flavanols, coumaric acids, isoflavones, hydrolyzable tannins, and derivatives of caffeic acid were among the classes of compounds found in the gum extract. Daidzein 4′‐O‐glucuronide was the main compound detected; other compounds found included 2,3‐digalloylglucose (Rt = 32.19 min; *m/z* 483), kaempferol diglucoside (Rt = 58.02 min; *m/z* 609), and quercetin‐pentoside‐hexoside (Rt = 43.24 min; *m/z* 595).

Dicaffeoylhexaric acid was detected at 32.12 min with *m/z* 533, and is recognized due to its anticancer, anti‐inflammatory, antiviral, and antioxidant abilities (Bourais et al. 2022; Tolba et al. 2016). Flavonols were represented by three main peaks belonging to quercetin‐rhamnosyl‐diglucoside (15.64 min), quercetin 3‐O‐rutinoside (17.26 min), and quercetin glucosyl‐glucuronide (18.13 min). Tannins were represented by ellagic‐glucoside at 18.67 min, *m/z* 463 (Al‐Khayri et al. 2022). Additionally, procyanidin B3, a compound widely recognized by its antioxidant properties, was detected at 16.87 min with *m/z* 577 (Zhuan et al. 2022). The detected compounds were identified based on data obtained in both ionization modes. For the aqueous extract of gum, several chemical classes were detected such as flavanols, coumaric acids, isoflavone, hydrolyzable tannins and caffeic acid derivatives, as shown in Figure 3 and Table 3.

**FIGURE 3:**
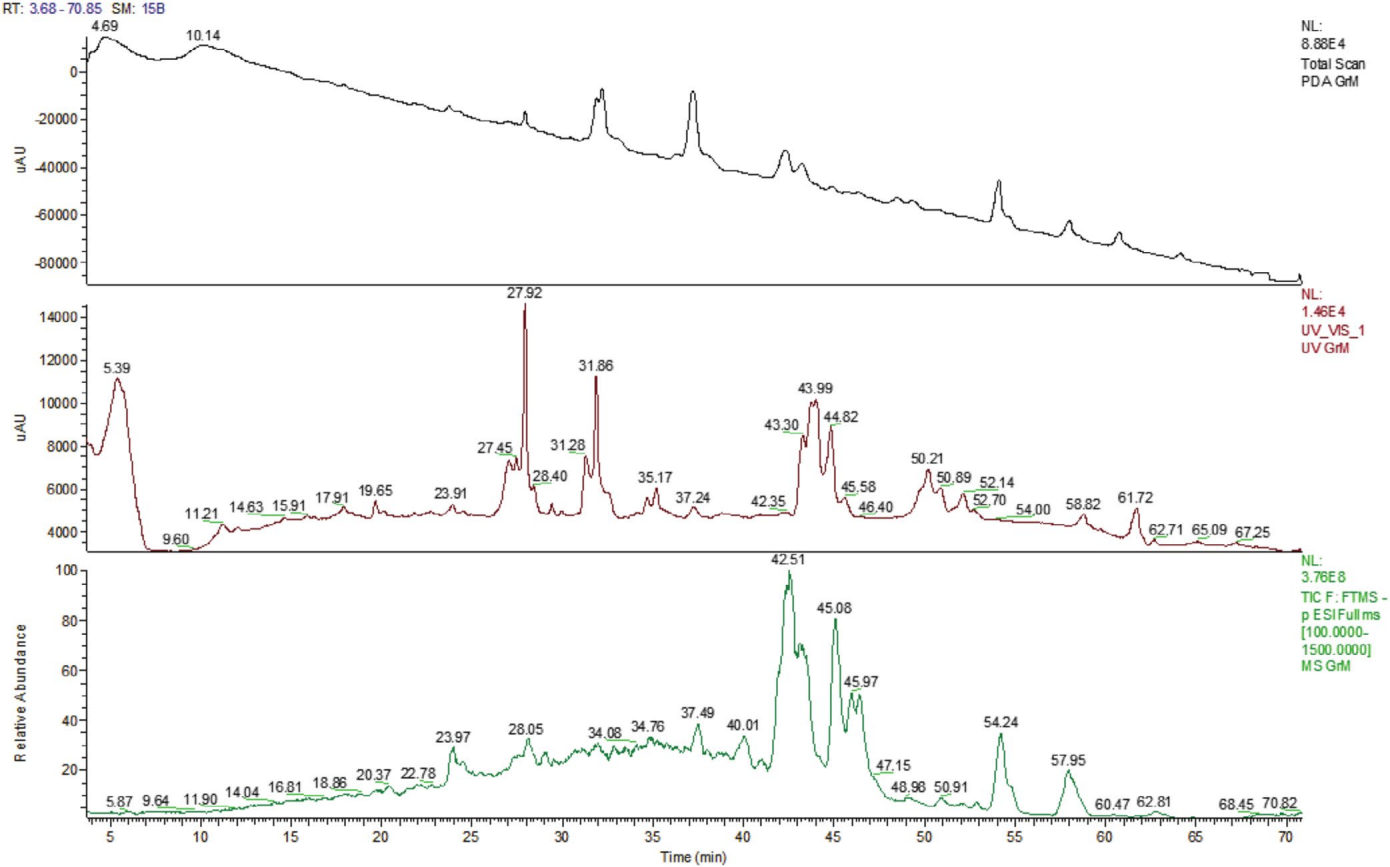
AEGL sample showing the LC‐DAD total chromatogram recorded between 190 and 400 nm, the LC‐DAD chromatogram recorded at 280 nm, and the total ion chromatogram (TIC) acquired in negative ion mode.

**TABLE 3:** Identification of phenolic compounds of AEGL sample by HPLC‐DAD‐ESI‐MS/MS.

No	Rt (min)	[M−H]^−^	MS/MS fragments (*m/z*)	Compounds	Chemical class
1	17.90	285	145/157/187/285	Kaempferol	Flavonols
2	27.92	383	197/331/303	O‐methyl‐epicatechin‐O‐sulfate (3)	Flavanols
3	31.87	527	143/223/325/339/397/527	Di‐caffeic acid derivatives	Caffeic acid derivatives
4	32.19	483	143/293/409/483	2,3‐Digalloylglucose	Tanins hydrolysables
5	37.23	429	309/349/411/429	Daidzein 4′‐O‐glucuronide	Isoflavone
6	42.27	409	293/311/347/409/	p‐Coumaroyl derivative	Coumaric acid
7	43.24	595	297/395/429/595	Quercetin‐pentoside‐hexoside	Flavonols
8	54.11	549	279/441/511/549	Quercetin‐3‐6‐malonyl‐glucoside	Flavonols
9	58.02	609	141/161/281/295/609	Kaempferoldiglycoside	Flavonols

The most abundant compound was daidzein 4′‐O‐glucuronide which belongs to the family of isoflavones and exhibits antioxidant, antimicrobial, anticancer, and anti‐inflammatory functions (Al‐Khayri et al. 2022). Peak 5 was identified as 2,3‐digalloylglucose (Rt = 32.19 min; *m/z* 483). Kaempferol diglucoside (Rt = 58.02 min; *m/z* 609) was also detected and is reported to possess potent antioxidant and anti‐inflammatory properties (Al‐Khayri et al. 2022).

This compound also has neuroprotective properties, reducing cellular damage through the inhibition of inflammatory pathways such as AKT, p38MAPK, and NF‐kB. Quercetin‐pentoside‐hexoside has a high therapeutical potential against cardiovascular disease especially by blood pressure lowering and inflammation inhibition (Al‐Khayri et al. 2022), this compound was detected at 43.24 min, *m/z* 595. In addition, di‐caffeic acid derivatives and p‐Coumaroyl derivative were detected respectively at 31.87 min with *m/z* 527 and at 42.27 with *m/z* 409.

The samples display a very intriguing bioactive diversity, and multiple distinct chemical classes including flavonols, hydrolyzable tannins, phenolic acids, carbohydrates, caffeic acid derivative, and coumaric acids, which make *V. tortilis* a rich source of active principle with several biological processes such as anti‐inflammatory, anticancer, antidiabetic, and anti‐oxidant aspects (Monteiro Espíndola et al. 2019; McDunn et al. 2013; Appendino et al. 2007).

The DPPH radical scavenging assay results presented in Table 4 demonstrated that extract L exhibited considerable antioxidant activity with an IC_50_ value of 0.032 ± 0.002 mg/mL, whereas no detectable activity was observed for extract G.

**TABLE 4:** Antioxidant activities of *Vachellia tortilis* extracts determined by DPPH, ABTS, and FRAP assays.

Extract/standard	DPPH (IC_50_ mg/mL)	ABTS (IC_50_ mg/mL)	FRAP (mg TE/g DW)
OLE	0.032 ± 0.002^a^	0.177 ± 0.002^a^	42.783 ± 0.301
AEGL	nd	nd	nd
Ascorbic acid	0.001 ± 0.0006^b^	—	—
Trolox	0.002 ± 0.0006^c^	0.012 ± 0.001^b^	—
BHT	0.003 ± 0.0006^d^	—	—

*Note:* Data are expressed as mean ± SD (*n* = 3). Values are expressed as mean ± SD (*n* = 3). Superscript letters within a column denote statistical grouping among the antioxidant reference standards only; extract (OLE) values are presented descriptively and were not included in inferential comparisons, as each extract was derived from a single extraction (technical replicates).

The antioxidant standards, including ascorbic acid, Trolox, and BHT, exhibited lower IC_50_ values than the plant extract, reflecting their higher antioxidant activity (Table 4).

The antioxidant potential of the extracts was evaluated using three complementary assays, DPPH, ABTS, and FRAP. The ABTS assay results summarized in Table 4 revealed that extract L possessed antioxidant activity with an IC_50_ value of 0.177 ± 0.002 mg/mL, whereas no detectable activity was observed for extract G.

The low IC_50_ value obtained for Trolox (0.012 ± 0.001 mg/mL) reflects its strong antioxidant capacity relative to the tested extract. The differences in antioxidant capacity between extract L, extract G, and the standard antioxidant are clearly represented in Figure 4.

**FIGURE 4:**
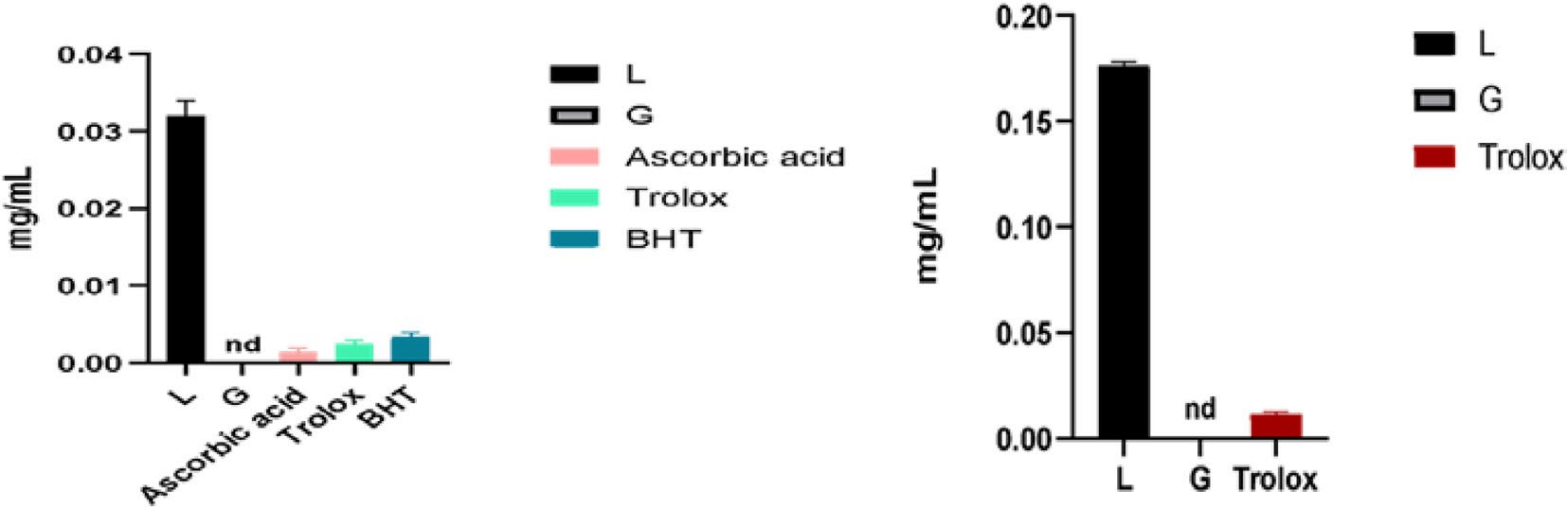
IC_50_ values of the DPPH and ABTS antioxidant activities of plant extracts and reference standards. OLE (leaf extract) and AEGL (gum extract) are represented as L and G, respectively.

According to Table 4, extract L demonstrated ferric reducing antioxidant power with a value of 42.783 ± 0.301 mg TE/g DW, whereas no detectable reducing activity was observed for extract G.

The reducing power observed in extract L indicates its capacity to donate electrons and reduce ferric ions (Fe^3+^) into ferrous ions (Fe^2+^). This activity may be associated with the elevated levels of polyphenols and flavonoids previously detected in the phytochemical screening.

The activity observed in the present study was lower than that reported previously (Alphonsine et al. 2019), using the DPPH assay, with an IC50 value of 0.03 ± 0.01 μg/mL. The disparities could be caused by changes in plant material, experimental or standard conditions, and extraction methods. In contrast, the lyophilized aqueous gum extract showed no detectable antioxidant activity, which is consistent with the findings of Ali and El Said (2020) who reported significant activity only at very high concentrations (92.13% ± 0.13% at 20 mg/mL).

Several studies have emphasized the vital role of phenolic compounds in the antioxidant mechanisms, both in vivo and in vitro (Frankowski et al. 2022). Furthermore, numerous earlier studies have shown an important correlation between phenolic concentration and antioxidant activity (Lefack Bongmo et al. 2022).

The results presented in Table 5 indicate that both extracts possess α‐amylase inhibitory activity, suggesting a potential antidiabetic effect. The IC_50_ values obtained for extracts L and G were 0.379 ± 0.002 mg/mL and 0.461 ± 0.001 mg/mL, respectively.

**TABLE 5:** α‐Amylase inhibition of *Vachellia tortilis* extracts.

Extract/standard	% of inhibition at 1 mg/mL	IC_50_ (mg/mL)
OLE	70.311 ± 1.573	0.379 ± 0.002
AEGL	42.101 ± 0.801	0.461 ± 0.001
Acarbose	80.401 ± 0.510	0.041 ± 0.0006

*Note:* Data are expressed as mean ± SD (*n* = 6).

The lower IC_50_ value recorded for extract L may reflect a higher capacity to inhibit α‐amylase activity. In comparison, acarbose, used as the reference drug, exhibited the lowest IC_50_ value (0.041 ± 0.0006 mg/mL), confirming its stronger inhibitory effect. As shown in Figure 5, both extracts were able to inhibit α‐amylase activity, although their effects remained less pronounced than that of acarbose.

**FIGURE 5:**
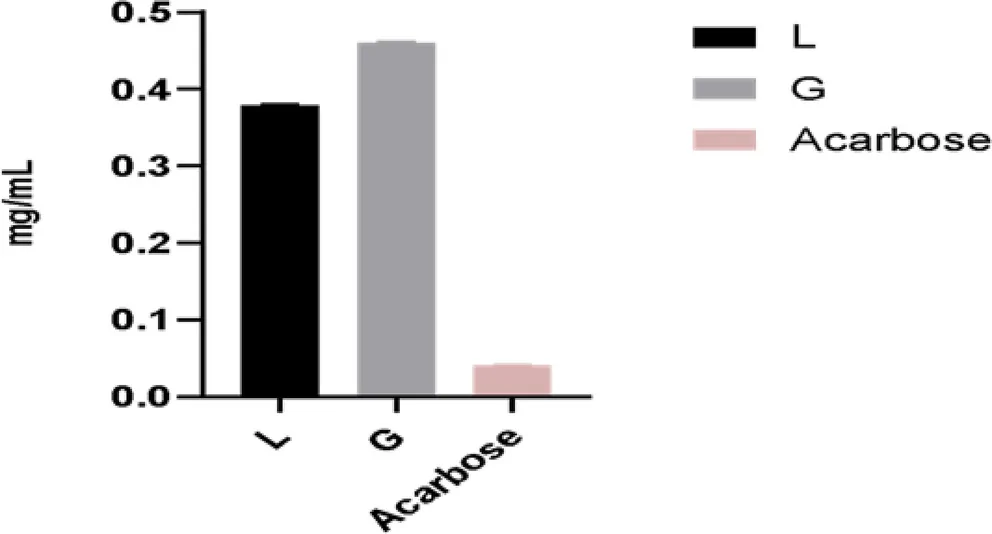
IC_50_ values of the α‐amylase inhibitory activity of both extracts and the reference standard. OLE (leaf extract) and AEGL (gum extract) are represented as L and G, respectively.

The α‐amylase inhibitory activity of the hydroethanolic leaf extract of *V. tortilis* (IC_50_ = 0.379 ± 0.002 mg/mL) was lower than that reported for *Acacia auriculiformis* , whose methanolic leaf extract exhibited a markedly stronger inhibitory effect (IC_50_ = 0.003 mg/mL) (Sharma et al. 2022). This comparison indicates that the hydroethanolic leaf extract of *V. tortilis* is less potent against α‐amylase than the methanolic leaf extract of *A. auriculiformis* .

Furthermore, considerable α‐amylase inhibition was shown by the hydroethanolic extract of *Acacia ligulata* leaves (Jaeger et al. 2018) and the alcoholic extract of *Acacia catechu* leaves (Khadayat et al. 2020), with IC_50_ values of 0.013 ± 0.007 and 0.049 ± 0.004 mg/mL, respectively. Although it exhibits somewhat less activity than previously mentioned Acacia species, these values highlight the strong inhibitory activity of the hydroethanolic extract of *V. tortilis* leaves.

Therefore, additional research including in vivo investigations and molecular mechanism analyses is required to fully comprehend the antidiabetic properties of these extracts. Aldahasi and Mohammed (Aldahasi and Mohammed 2025) state that the species' potential as a source of bioactive compounds for pharmaceutical and nutraceutical substances is further supported by advancing biotechnological interest in *V. tortilis* .

Previous research has highlighted the role of phenolic compounds in influencing glucose metabolism. These compounds have been shown to improve insulin sensitivity, regulate intestinal glucose absorption, and reduce blood glucose levels (Ávila‐Román et al. 2021). Nevertheless, the α‐amylase inhibition assay represents only one aspect of the complex mechanisms involved in glucose regulation, despite suggesting potential antidiabetic activity. A limitation of the present study is that each extract was prepared from a single extraction. Therefore, the findings should be considered preliminary and confirmed in future studies using independent extraction replicates. Further studies, including in vivo investigations and molecular analyses, are also needed to validate and better elucidate the antidiabetic properties of *V. tortilis* extracts.

## 4 Conclusion

This preliminary study evaluated the polyphenol compound, antioxidant capacity, and inhibitory effects on digestive enzymes of *V. tortilis* leaf and gum extracts. Our study is the first to show the α‐amylase inhibitory properties of *V. tortilis* , with emphasis on HPLC‐DAD‐ESI‐MS/MS profiling. Interestingly, the hydroethanolic extract showed strong antioxidant potential as shown by DPPH, FRAP, and ABTS assays, in addition to its strong inhibitory effect on the α‐amylase enzyme. These results imply that polar extracts from the underappreciated Moroccan plant *V. tortilis* may have therapeutic relevance in the treatment of Type 2 diabetes and the reduction of oxidative stress. More investigation is necessary to explore the pharmaceutical applications of these bioactive substances through advanced biological studies. Future investigations should also focus on the molecular docking of this species to elucidate their potential in treating a wide range of health conditions.

## Author Contributions

**Douae Taha:** conceptualization, writing – original draft, data curation, methodology. **Souad El Hajjaji:** conceptualization, methodology, writing – review and editing, supervision. **Yassine Mourabit:** data curation, writing – review and editing. **Waleed Al Abdulmonem:** funding acquisition, investigation, writing – review and editing, visualization. **Mohammed Alorini:** funding acquisition, visualization, writing – review and editing, formal analysis. **Abdelhakim Bouyahya:** investigation, methodology, writing – review and editing, formal analysis, supervision. **Naima Iba:** conceptualization, writing – review and editing, methodology. **Ilhame Bourais:** conceptualization, methodology, supervision, writing – review and editing.

## Funding

This study was supported by the Deanship of Graduate Studies and Scientific Research at Qassim University (http://www.qu.edu.sa) for financial support (QU‐APC‐2026).

## Conflicts of Interest

The authors declare no conflicts of interest.

## Data Availability

The data that support the findings of this study are available from the corresponding author upon reasonable request.
